# Relationships Among Activity, Motor Skill Performance and Executive Function in Preschool Children: Observational Report

**DOI:** 10.1111/cch.70263

**Published:** 2026-03

**Authors:** Alyssa M. Button, Ethan Abbenante, Robbie Beyl, E. Kipling Webster, Anthony Okely, Amanda E. Staiano

**Affiliations:** 1 Pennington Biomedical Research Center, Baton Rouge, Louisiana, USA; 2 Virginia Commonwealth University, Richmond, Virginia, USA; 3 University of Tennessee, Knoxville, Tennessee, USA; 4 University of Wollongong, Wollongong, Australia

**Keywords:** child development, inhibitory control, physical activity, sedentary behaviour, sleep, visual–spatial working memory

## Abstract

**Background:**

Executive functions are important for navigating daily demands and engaging in goal-driven behaviour. While these functions are associated with motor skills and activity in mid-to-late childhood, there is a paucity of available data among preschool ages. We hypothesized that child activity, motor skills and executive function would be associated among preschool-aged children.

**Methods:**

Children aged 3–4 years wore an Actigraph GT3X+ for 7 days to assess activity levels. Product-oriented motor skills were assessed that measure balance, manual dexterity, agility, strength and coordination. The Early Years Toolbox was used to measure visual–spatial working memory via the Mr. Ant Task and inhibitory control using the Go/No-Go task. Pearson correlations evaluated associations among both physical activity and motor skill performance with executive function. *N* = 83 children provided data for the analytic sample.

**Results:**

Positive relationships (*p* < 0.001) were observed among inhibitory control and visual–spatial working memory and standing long jump (*r* = 0.46, *r* = 0.28), one-legged balance (*r* = 0.36, *r* = 0.40) and grip strength (*r* = 0.41, *r* = 0.44). A negative relationship (*p* < 0.001) was observed between quicker STUG times and inhibitory control and visual–spatial working memory (*r* = −0.47, *r* = −0.48).

**Conclusions:**

The bidirectional associations of motor competence and executive function are evident during this rapid time of development. Cognitive functions are required for concerted movement and inhibiting nongoal-directed movements, indicating that this is an important period to provide ample opportunities for learning.

## 1 | Introduction

The early years of childhood are marked by significant physical and cognitive growth (Carson et al. 2017). Among preschool-age children (ages 4–6 years) (Kail 2011), key cognitive functions like inhibitory control (IC) and visual–spatial working memory (VSWM) are crucial for safely and efficiently navigating daily demands. IC requires the ability to regulate attention, behaviour and emotions (Horvath et al. 2022). While IC is fundamental for goal-directed activities ranging from walking safely to using scissors, the relationship among physical activity (PA), motor skills and IC in preschoolers remains unclear (Moradi et al. 2019; Zhang et al. 2024). Similarly, VSWM, which manages spatial information, is essential for motor planning and coordination (Zheng et al. 2023). Recent research suggests that these functions share a common working memory system (Tiego et al. 2018). Between the ages 3 and 5 years, children undergo remarkable progression in executive functioning, including IC and VSWM, making this a critical window for cognitive growth (Carlson 2016).

PA and movement behaviours (PA, sedentary activity and sleep) are potential contributors to cognitive development (e.g., IC and VSWM), though evidence is mixed (Bidzan-Bluma and Lipowska 2018; Gunnell et al. 2019). While systematic reviews in older children show small to large positive correlations between PA and cognitive performance, large-scale randomized trials have found no definitive evidence of exceptional benefit (Gunnell et al. 2019). According to the World Health Organization (WHO) guidelines (World Health Organization 2019) preschool children should engage in 180 min of daily PA (60 min of moderate to vigorous PA; MVPA), and 10–13 h of quality sleep (Tremblay et al. 2017). Young children, ages 3–6 years, who achieve the recommended hours of 24-h sleep for their age demonstrate better IC and working memory abilities (Nieto et al. 2022; Ltifi et al. 2025). Moreover, recent reports show a decline in PA and an increase in sedentary behaviour as children age (Hayes et al. 2019). Approximately 60% of preschool-aged children are reported as meeting the WHO guidelines (Bourke et al. 2023), yet only 26%–42% achieve these amounts between ages 6 and 11 (Carlson et al. 2025).

Developing motor skills is a cornerstone of health child development, creating a foundation for both PA and cognitive growth (Bondi et al. 2022). Gross motor skills encompass object control and locomotor skills considered necessary for application in context-specific sports, games and PA (Logan et al. 2018). Among children ages 4–6 years old, inverse correlations are evident between gross motor skills and the reaction component of IC (Liu et al. 2022). Using the Fish Flanker Task, Liu and colleagues concluded that better gross motor skills according to the Test for Gross Motor Development (2nd edition, TGMD-2) were associated with quicker time choosing the correct response. Others have found that as gross motor skills improve (measured via Korper Koordinationstest für Kinder and Bruininks-Oseretsky Test of Motor Proficiency), VSWM skills (measured via adapted spatial span task) also improved within a sample of 8–10-year-olds (van der Fels et al. 2020). In children aged 10–12 years, a positive correlation between motor ability and academic achievement was discovered, which was determined to be mediated by executive function skills, including inhibition (Oberer et al. 2018; Schmidt et al. 2017). This relationship is often explained by the theory of embodied cognition, which suggests that as children learn to move and interact with their environment, they physically strengthen the neural pathways required for higher-order cognition (Gibbs 2005; Harbourne and Berger 2019; Klupp et al. 2021).

Engagement in PA is positively associated with improvements in motor skills, with emerging evidence for similar findings among PA engagement and cognitive abilities such as working memory in children ages 4–6 (Zeng et al. 2017). While there is robust evidence of a positive correlational relationship between cognitive function, specifically IC, VSWM and PA across older age cohorts, there is a notable gap in the literature when it comes to preschool-aged children (Nieto et al. 2022; Serra et al. 2021). Developmental differences mean these findings cannot be assumed to generalize to preschool-aged children, i.e., the timing, mechanisms and magnitude of PA’s effects likely differ (Oberer et al. 2018). Investigations of early childhood IC and VSWM may be understudied compared to older aged cohorts due to the difficulty of standard executive function tasks for this age group (Zheng et al. 2023), thus the use of adapted tools may be justified. These methodological barriers have created a notable gap in understanding how PA relates to IC and VSWM during early childhood. Understanding the relationships among these variables during this early stage may clarify opportunities to enhance the timing, mechanisms and magnitude of PA to support cognitive processes (i.e., IC and VSWM) involved in long-term academic and health outcomes.

The aim of the current study was to fill this knowledge gap by examining how movement behaviours (PA and sleep) and motor skill performance (fine and gross) were associated with executive function outcomes (i.e., IC and VSWM) among preschoolaged children. The primary hypothesis is that children with stronger IC and VSWM will demonstrate better motor skill performance and greater adherence to recommended movement behaviour guidelines. Conversely, better motor skill competence and healthier movement behaviours are expected to be associated with stronger executive function performance.

## 2 | Methods

### 2.1 | Participants

This dataset is a southern US subset of the observational SUNRISE study, an international examination of movement behaviours that aims to measure PA, sedentary behaviour and sleep for children under the age of five (Okely et al. 2021). Participants were recruited from the southern US region via email listservs, social media, word of mouth and flyers at local childcare centres. Eligibility criteria included children between the ages of 3 and 4 years old, and parents’ ability to read and/or speak English proficiently. Exclusion criteria included parent-report of child mobility limitations.

### 2.2 | Procedures

Parents interested in the study were screened using a web-screener and follow-up phone call. After the phone screening, Visit 1 was scheduled for in-person assessment at either the research facility or their child’s early childhood education centre. At Visit 1, parents provided consent, completed surveys and the child completed height and weight measurements, received an accelerometer device and completed motor skills and cognitive tests. Testing conditions (e.g., surface, space and equipment) were standardized using a manual of procedures to reduce environmental variability. The accelerometer was returned to the research centre or mailed back via a premetred envelope after approximately 1 week of wear. Participants who completed the visit received a small toy as compensation (estimated to be $5/child). Once the accelerometer was received by the study team, the parent was mailed a $25 check. All procedures were approved by the SUNRISE Coordinating Center with written ethics approval from the local institutional review board (Pennington Biomedical Center, FWA #00006218) in compliance with relevant laws and institutional guidelines.

*PA and sedentary behaviour* were measured using a triaxial accelerometer (Actigraph GT3X+, Ft. Walton Beach, FL). Each child wore the ActiGraph continuously for 7 days (with the exception of time spent in water, e.g., while bathing) on their right hip. Data were processed using ActiLife software version 6.12.1 and divided into 15-s epochs for analysis. Non-wear time was defined as 20 min of consecutive 0 counts. Data were included if the child had at least 5 days of accelerometry data, with at least 6 h of wear time per day as done in other studies (Bingham et al. 2016; Chong et al. 2024). *Sleep* was estimated via total active wear minus parent-reported minutes of sleep (based on typical bed, wake and nap times).

*Motor skills* were assessed using a range of product-oriented measures. To measure upper body strength, a hand dynamometer was used to assess grip strength, measured to the closest 0.1 kgf. A nine-hole pegboard was used to measure manual dexterity and hand manipulation, where children were timed (seconds) picking up nine pegs one at a time and inserting them into a 31.1 cm × 26.0 cm board and then replacing the pegs back to the original position. This measure is valid and reliable for use in preschool-aged children (de Vries et al. 2015).

Children performed three tests of gross motor skills including supine timed up and go (STUG), used to measure mobility and coordination. To complete this skill, children lied supine with the heels of their feet on a line 3 m away from a wall; when instructed ‘go’, children stood up and raced to touch a target marked on the wall and then turned and ran as quickly as they could over the starting line. This skill was measured in seconds, with a shorter amount of time indicating better performance.

The next skill, the one-legged standing balance test, measured posture and balance. Children stood on one leg for as long as possible. Performance was measured in seconds, with longer times (up to 30 s) indicating better performance. The third test of gross motor skills was the standing long jump, which measured explosive leg strength and mobility. Children stood with their feet on a line marked on the floor, and then jumped as far as possible with two feet together. Performance was measured as the furthest distance (in cm). These skills each included one practice trial and two attempts, with attempt scores averaged for analyses.

*Executive function* was assessed using the Early Years Toolbox (EYT) (Howard and Melhuish 2017). EYT ‘Mr. Ant’ Task is an iPad-based assessment of VSWM. Children were presented with an image of a cartoon character—Mr. Ant—who has several coloured dots placed in different spatial locations on his body. After 5 s, these dots disappeared, a blank screen was presented for 4 s, and then the child was asked to recall the locations for the dots by tapping on the corresponding locations on an image of Mr. Ant without stickers. Participants were provided with verbal instructions and three practice trials to become familiar with the task. There were three trials that increase in difficulty as the trial progresses from one to eight stickers. The trial continued until completion of all eight stickers in each trial, or failure on all three trials at the same level of difficulty, with points scored as one point for each trial in which at least two of three tasks were performed accurately, plus 1/3 of a point for all correct tasks thereafter. Performance was calculated based on a point score, which accounted for the number of levels completed and number of correct trials. EYT Go/No-Go is an iPad-based assessment of inhibition. Children were presented with fish and sharks for 1500 ms, separated by a 1000 ms interstimulus interval, and given the instruction to tap the iPad screen whenever they saw a fish, and to refrain from responding when a shark appeared. Children were provided instructions on completing the ‘go’ and ‘no-go’ demands and a block of 10 practice trials before beginning the task. Performance was calculated based on the proportion of accurate responses to selecting (or not selecting) the fish. For each of these tests of executive function, a comparison of performance to developmental expectations, based on a normative sample of children of the same age and gender was produced (Howard and Melhuish 2017).

*Child activity and demographics* were assessed via parentreport questionnaire. Questions about activity collected information related to child sedentary time, travel time, screen time and sleep based on each behaviour guideline (Okely et al. 2017). Questions about parent and child demographics were based on the WHO STEPwise Survey (Organization WH 2017).

### 2.3 | Analysis

The sample size of this study was determined to detect 80% power and a 5% significance level to detect small effects (Okely et al. 2021). Prior to analyses, data were checked for normality. Pearson correlations were used to evaluate the associations among both activity and motor skill scores with performance on executive function tasks. Chi-square analyses were used to evaluate differences in whether children met or did not meet PA guidelines based on performance on executive function tasks based on developmental expectations by age in years and months compared to a normative sample of 1764 same age peers (i.e., above age expectations [75th percentile], somewhat below age expectations [25th percentile] and within age expectations [50th percentile]) (Howard and Melhuish 2017). Data may be made available upon reasonable request. This study was not preregistered.

## 3 | Results

A total of 106 children were recruited for this regional sample within the larger, international study (*n* = 50 [47%] girls, *n* = 56 [53%] boys; *n* = 62 [70%] White, *n* = 15 [17%] Black, *n* = 9 [10%] Asian, *n* = 3 [3%] other; *n* = 85 [96%] not of Hispanic/Latino/a or Spanish Origin; *n* = 99 [93%] living in an urban setting). Of those, 83 had complete data that included accelerometer wear (missing = 23), executive function task performance (missing = 8) and motor skill performance (missing = 11). The majority of the sample met the recommended WHO guidelines for sleep (*n* = 93, 89%) and moderate to vigorous PA (*n* = 69, 83%). Less than half the children (*n* = 49, 47%) met the sedentary time recommendations (World Health Organization 2019). Table 1 shows the demographic and descriptive data.

Pearson correlations revealed no significant associations between movement behaviours and executive functions (Table 2). Performance on the motor skill tasks had small to medium effects (Cohen 2013) (*p* < 0.05) with executive functions, with the exception of the nine-hole pegboard. IC and VSWM were each associated with standing long jump (*r* = 0.46, *r* = 0.28), onelegged balance (*r* = 0.36, *r* = 0.40) and grip strength (*r* = 0.41, *r* = 0.44), where improved cognitive performance was associated with improved motor skills. Similarly, better STUG performance was associated with greater IC and VSWM scores (*r* = −0.47, *r* = −0.48). These values are presented in Table 3. Chi-square tests evaluating the relationships between activity guidelines and executive function did not reveal significant differences (Table 4).

## 4 | Discussion

The present study evaluated functional motor skill performance, executive function and time engaged in PA, sedentary and sleep in preschool-age children. No associations were observed among executive function and movement behaviours. As performance on IC and VSWM tasks increased, so did performance on most of the motor skills assessed, including the standing long jump, one-legged balance, grip strength and STUG tasks. This study was innovative in that it provides evidence of connections and interactions among executive function and motor skills in children as young as 3 years old, which is earlier than what many previous studies have shown (van der Fels et al. 2020; Oberer et al. 2018; Schmidt et al. 2017).

Executive function tasks in this study were not associated with children’s movement, including sleep. While some research indicates a positive relationship between early childhood sleep and executive functions, particularly in older children (Bernier et al. 2021; Chen et al. 2021), others suggest sleep quality, rather than quantity, is more crucial at this developmental stage (Zhang et al. 2021). The relationship between executive function and sedentary behaviour requires more nuanced understanding, as accelerometer-based measurements may not capture essential contextual information (Bezerra et al. 2024). Findings from the current study contribute to the heterogeneity of available results, with mixed support for cross-sectional associations between sleep, MVPA and sedentary behaviour (Carson et al. 2017; Luo et al. 2023). Although increased MVPA is associated with improved cognitive performance in young children, most evidence excludes spatial and memory domains, where IC and VSWM are dominant (Carson et al. 2017). While MVPA was not associated with IC or VSWM in the current study, other research suggests that cognitively enriched PA may improve both cognitive and motor skills in 3–6 year olds (Biino et al. 2023), suggesting a potential relationship between early MVPA and later cognitive outcomes. We hypothesize that PA measurements in the present sample may have captured general domains of play that are not as cognitively demanding as those employed in enrichment trials (Maurer and Roebers 2019). Understanding the differences among IC, VSWM and PA based on cognitive complexity of the activity may be a fruitful endeavour in future work to inform intervention development.

The relationships observed between motor skills and executive function are supported by the developmental theory of parallel development, which posits that children with better visual-motor coordination are more likely to manipulate objects with their hands, a requirement for tasks that also require the use of cognitive processes. Others have identified a link between IC and motor skills and VSWM and motor skills in older age groups (Maurer and Roebers 2019), but this is among the first studies to demonstrate these relationships in 3–4-year-old children (Iivonen and Sääkslahti 2014). The current study extends this work by suggesting that executive function, specifically IC and VSWM, may also be relevant factors to consider as potential determinants.

Grip strength and gross motor skill performance were positively related with executive function performance, suggesting that individual differences in higher-order cognitive control are already observable in early childhood. Instructions for some of these tasks are multistep, requiring the children to attend to instructions and retain information for later use. Gandolfi et al. studied performance of children on an IC task in two distinct conditions: response inhibition with low working memory demands, and response inhibition with interference suppression (associated with higher working memory demands). Findings showed that children ages 36–48 months are developing distinguished IC processes that are more complex than those of younger, indicating this as a key transition point in children’s development of IC (Gandolfi et al. 2014). The associations between gross motor skills and advanced IC processes are evident in the current sample. Looking ahead to the next developmental stage for children between ages 5 and 6, executive function was well-established as associated with gross motor skills only when these tasks were complex, i.e., less automated and requiring more cognitive demand (Maurer and Roebers 2019). Based on the tremendous cognitive development occurring in the first few years of life, PA curricula for preschoolers should be intentionally designed to include complex, nonautomated tasks requiring attention and information retention, leveraging motor development as a conduit for enhancing early executive function.

The findings of this study are limited in the following ways. First, generalizability is limited due to the relatively racially and economically homogenous sample. Due to English-only language inclusion criteria and voluntary participation, selection bias may be a limitation of this study. Larger explorations of these variables are underway among geographically and economically diverse samples, which will advance our abilities to make broader conclusions across diverse populations (Okely et al. 2021). Further, there was a sizeable portion of the sample that had missing accelerometer data, potentially leading to less power to detect relationships. Given that cognitive, motor and PA skills are rapidly developing and associating at different times across early and mid-childhood, research may benefit from understanding developmentally appropriate measures and interventions for brief and discrete periods of time to provide optimal effectiveness and utility. Additionally, different motor skill assessments (e.g., process oriented), or ones that are more multifaceted product-oriented batteries, like Movement Assessment Battery for Children—Third Edition (Wu et al. 2025), which assesses four fine motor skill tasks, may provide additional information and mechanistic clarification for these relationships. Finally, results of the study may be limited by possible recall or social desirability bias, particularly for parent-reported sleep.

The bidirectional associations of motor competence and executive function are evident early on in life among children ages 3–4 years during a time of rapid development. This may be an important period to provide opportunities for learning and practice in order to optimize development for later positive outcomes associated with these early fundamental skills. These results can inform the efforts of educators, policymakers and clinicians to prioritize multistep activities, redefine ‘active time’ in curricula, and utilize gross motor skills as early indicators of cognitive health.

## Figures and Tables

**TABLE 1 |:** Demographic and scores descriptive table.

Demographics	*N*	%	Mean	SD

Boys	56	53		
Girls	50	47		
Race				
Asian	9	10		
Black or African American	15	17		
Other	3	3		
White	62	70		
Ethnicity				
Hispanic Mexican, Mexican American or Chicano/a	1	1		
Hispanic Puerto Rican	1	1		
Not of Hispanic, Latino/a or Spanish origin	85	96		
Other Hispanic, Latino or Spanish origin	2	2		
Sector				
Urban	99	93		
Rural	7	7		
Parental education				
High school/GED	7	7		
Associate’s degree or 1–3 years of college	11	10		
Bachelor’s degree	28	27		
Graduate/professional degree	59	56		
Food security status				
Secure	102	97		
Insecure	3	3		
Child age	105		3.9	0.60
BMIz	105		0.58	1.01
Variable measurements	*N*	%	Mean	SD
Sleep (minutes)	83		709.93	133.97
Sedentary activity (minutes)	83		463.21	55.95
MVPA (minutes)	83		86.50	26.61
Go/no-go	99		0.51	0.19
Mr. Ant				
Point score	98		1.03	0.93
Supine timed up and go average (seconds)	99.5		6.07	1.48
Standing long jump	102		53.33	20.16
One-legged balance (right)	101		5.21	5.54
One-legged balance (left)	100		5.15	5.59
Nine-hole pegboard (seconds, right)	96		37.12	14.97
Nine-hole pegboard (seconds, left)	95		33.79	18.20
Grip strength (right)	89		5.92	2.55
Grip strength (left)	89		5.63	2.60
Meeting physical activity guidelines	*N*	%	Mean	SD
Sleep (minutes)	93	89		
Sedentary activity (minutes)	49	47		
MVPA (minutes)	69	83		
Go-no/go development status	*N*	%	Mean	SD
Above age expectations	20	20		
Somewhat below age expectations	7	7		
Within age expectations	72	71		
Incomplete assessment	2	2		
Mr. Ant development status	*N*	%	Mean	SD
Above age expectations	18	18		
Somewhat below age expectations	6	6		
Within age expectations	74	76		

Abbreviations: BMIz = body mass index z-score, MVPA = moderate to vigorous physical activity.

**TABLE 2 |:** Correlations of movement behaviour (minutes) with executive function task performance.

	Go/no-go			Mr. Ant		
	N^a^	r	p	N	r	p
Sleep	80	−0.05	0.684	80	−0.11	0.327
Sedentary activity	80	−0.00009	0.999	80	0.19	0.100
MVPA	80	−0.15	0.199	80	0.09	0.442

Abbreviation: MVPA = moderate to vigorous physical activity.

a Analytic sample is less than the study sample due to missing data from incomplete assessments and accelerometer data.

**TABLE 3 |:** Correlations of motor skill performance with executive function task performance.

	Go-no/go			Mr. Ant		
	N	r	p	N	r	p
Supine timed up and go	96	−0.47	< 0.0001	96	−0.48	< 0.0001
Standing long jump	99	0.46	< 0.0001	98	0.28	0.006
One-legged balance (right)	98	0.36	0.0003	97	0.40	< 0.0001
One-legged balance (left)	97	0.27	0.0064	97	0.30	0.0036
Nine-hole pegboard	93	−0.04	0.736	95	0.03	0.767
Grip strength	85	0.41	< 0.0001	85	0.44	< 0.0001

**TABLE 4 |:** Executive function task performance among children who did or did not meet movement behaviour World Health Organization guidelines (Nieto et al. 2022).

	MVPA^a^				Sleep^b^				Sedentary activity^c^			
	Met (%)	Not met (%)	*X* ^2^	*p*	Met (%)	Not met (%)	*X* ^2^	*p*	Met (%)	Not met (%)	*X* ^2^	*^p^*
**Go-no/go**			0.73	0.694			0.97	0.616			0.35	0.840
Above age expectations	18	5			19	1			9	11		
Within age expectations	61	10			64	9			33	39		
Somewhat below age expectations	5	1			6	1			4	3		
**Mr. Ant**			2.61	0.271			3.87	0.144			0.56	0.756
Above age expectations	16	3			16	2			9	9		
Within age expectations	65	10			69	6			37	39		
Somewhat below age expectations	4	3			4	2			2	4		

Abbreviation: MVPA = moderate to vigorous physical activity.

a WHO MVPA recommendation: spend at least 180 min in a variety of types of physical activities at any intensity, of which at least 60 min is moderate- to vigorous-intensity physical activity, spread throughout the day; more is better.

b WHO sleep recommendation: have 10–13 h of good quality sleep, which may include a nap, with regular sleep and wake-up times.

c WHO sedentary activity recommendation: not be restrained for more than 1 h at a time (e.g., prams/strollers) or sit for extended periods of time. Sedentary screen time should be no more than 1 h; less is better.

## Data Availability

Data may be made available upon reasonable request.

## Summary

- Motor skills and executive functions, like inhibitory control and visual–spatial working memory, are bidirectionally associated as early as 3 years old; thus, practitioners should integrate training to optimize skill development simultaneously. Combined motor and cognitive tasks should prioritize complex movements requiring children to attend to instructions and retain information (e.g., obstacle courses with specific rules).
- Policymakers and educational boards should consider moving beyond total minutes of physical activity to quality and complexity of activity, incentivizing curricula that integrate cognitive challenges into physical education.
- Gross motor skill assessments should be considered as early indicators of cognitive health easily integrated into preschool physical wellness checks.
- Future research must move beyond accelerometers to understand the content of sedentary time—‘quiet time’ spent on cognitively demanding tasks may have different implications for executive function than passive screen time.
